# Transactions Vermont Medical Society, 1884

**Published:** 1885-07

**Authors:** 


TRANSACTIONS OF THE VERMONT MEDICAL SOCIETY, for the year 1884. Published by the Society.

This work contains a number of valuable papers, which are worthy of a better dress than this publication gives them. Nothing adds more to a work than a handsome binding, and this our friends of the Vermont Society should have for their future publications.

